# Obstructive Sleep Apnea and Cardiovascular Morbidities: A Review Article

**DOI:** 10.7759/cureus.10424

**Published:** 2020-09-13

**Authors:** Dibyata Rana, Chenet Torrilus, Wiqas Ahmad, Nkechi A Okam, Tehreem Fatima, Nusrat Jahan

**Affiliations:** 1 Internal Medicine, California Institute of Behavioral Neurosciences & Psychology, Fairfield, USA

**Keywords:** osa, cardiovascular diseases, pathology, biomarkers

## Abstract

In obstructive sleep apnea (OSA), there are brief episodes of partial or total upper airway obstruction during sleep, which leads to apnea or hypopneas. Much attention is required to understand OSA's effects on the human body, owing to how common but under-diagnosed this disorder remains. Though the role of OSA in cardiovascular (CV) disease is commonly discussed, it remains unclear how it induces changes in the human body. The intermittent and recurrent hypoxia occurring at the cellular level in this condition is critical for the dramatic changes observed. Vascular endothelial cell (VEC) injury and other mechanisms seen in OSA lead to changes in the CV system. OSA can take a toll on a person's overall functioning, especially with so much importance in today's time on preventing and treating cardiac-related deaths. A total of 31 published articles were included from the PubMed database for our literature review. Most of the studies showed a strong association of OSA with hypertension, especially resistant hypertension. Findings were consistent with OSA's independent role in causing CV diseases, included heart failure, coronary artery disease (cardiac ischemia), arrhythmias, and ischemic stroke. Continuous Positive Airway Pressure (CPAP) is one of the reliable and beneficial treatments for OSA patients. OSA is a treatable and modifiable risk factor for cardiac events and related deaths. The primary purpose of our review article was to address any existing gaps between OSA and its effect on the human body with particular emphasis on cardiovascular changes.

## Introduction and background

Like William Shakespeare quotes, "Innocent sleep. Sleep that soothes away all our worries. Sleep that puts each day to rest. Sleep that relieves the weary laborer and heals hurt minds. Sleep, the main course in life's feast, and the most nourishing." [1]. Sleep is a vital component of human life. When we put our body to rest, there is an occurrence of numerous cellular mechanisms during sleep. The cells of the human body repair, regenerate, and revitalize themselves to prepare for the next day to put our bodies into motion and continue with our daily lives. Sleep disorders are prevalent these days, and many of them significantly impact a person's overall well-being. One such common condition is sleep apnea, which is broadly classified into two types: central and obstructive, with the latter being the most common type. Obstructive sleep apnea (OSA) is characterized by repetitive collapse of the upper airway during sleep leading to a temporary cessation of breathing (apnea), shallow breathing (hypopnea), or respiratory-related arousals. OSA is further classified depending on the number of apnea and hypopnea episodes occurring per hour of sleep, commonly known as the Apnea-Hypopnea Index (AHI). Hence, OSA can be mild (5 ≤ AHI < 15), moderate (15 ≤ AHI < 30), or severe (30 ≤ AHI) [2]. OSA is among the prevalent sleep disorders associated with various health hazards, but unfortunately, this condition remains under-diagnosed.

Quite often, OSA remains unnoticed by the patient, and it is usually that their bed partner complains to them of loud snoring, snorting, choking, or cessation of respiration while they are asleep. A survey on OSA's current prevalence indicates that one-third of sleep studies showed some degree of OSA (AHI ≥5 events per hour of sleep). Nearly 13% of men and 6% of women between 30 and 70 have moderate to severe forms of OSA (AHI ≥15) [3]. There has been a substantial increase to millions of new sufferers with the ongoing unprecedented epidemic of obesity. About 50-60% of people who have obesity and metabolic syndrome have OSA [4,5], which could be a confounding factor in the cardiovascular burden effect seen commonly with obesity and metabolic syndrome [6]. Studies have now shown that OSA can independently increase cardiac risk by causing numerous cardio-metabolic dysregulations [7-9].

OSA is the most common secondary cause and worsening of hypertension despite adequate anti-hypertensive medications [10]. A comparative study by Pedrosa et al. [11] found OSA in more than 50% of the study population who had resistant hypertension, thus concluding that OSA is the primary cause for resistant hypertension (uncontrolled blood pressure (BP) with ≥three anti-hypertensive medications). Even in the setting of diagnosing OSA as a cause of resistant hypertension, the clinicians often tend to neglect this condition, when in fact, OSA has the potential to cause other cardiovascular morbidities. Various studies have found that OSA is significantly associated with other CV events such as ischemic stroke [12], myocardial infarction [13], heart failure, and arrhythmias [9]. Even with so many past and ongoing researches about CV health, deaths due to cardiovascular disease still tops worldwide. OSA is one such condition that can hamper CV health and modify the prognosis of any underlying cardiac diseases. Hence appropriate diagnosis and management of OSA, which is possible, can significantly improve the cardiovascular morbidity and mortality in a patient. Our literature review article emphasizes on the independent association of OSA with various cardiovascular dysregulation and the mechanism behind it.

## Review

Method

The central electronic databases used were MEDLINE/PubMed and PubMed Central (PMC), including the terms of Medical Subject Heading (MeSH). 'Sleep apnea' and 'Hypertension' were the two MeSH words used together. Our investigation included studies on humans of 45-64 years, published in the last 10 years and in the English language. Gender differences were not taken into account. Studies in non-English language and performed on animals were excluded for this review. Our study was primarily targeted to find sleep apnea as an independent cardiovascular risk factor. We have worked as a team and divided members into two groups. Group one included three members, and group two included two members. Group two was assigned to read through all the titles and the abstract while group one read through all the free full-text articles' information. The information provided in those publications was recorded manually on Microsoft Excel sheets; this included the publication title, journal name, date of publication, Digital Object Identifier (DOI), abstract, and full-text information. Each detail on the datasheet was again read carefully by the team members to annotate points in a different datasheet that supported our research objective. As this was a traditional literature review, Quality Assessments of the studies and approval by the Research and Ethics Committee were not done.

Result

A total of 31 articles were gathered for this literature review. Twenty-seven articles were free full-text publications, whereas the remaining four articles were only abstracts.

'Sleep apnea' and 'Hypertension' in the MeSH search builder yielded 2094 articles. Five hundred thirty-nine publications remained with the inclusion criteria of human studies, age group 45-64 years, English language, and published within 10 years. Exclusion criteria included animal studies and non-English literature. The finding is summarized in the following table (Table 1).

**Table 1:** MeSH keywords used to find relevant studies for the literature review MeSH, Medical Subject Headings; No., Number

MeSH Search for MeSH keywords 'Sleep apnea' And 'Hypertension.'	No. of records
Total record	2094
After applying inclusion and exclusion criteria	
Humans	2052
Age 45-64 years	1071
English language literature	941
Published within ten years	539
Free full-text literature	241

**Figure 1:**
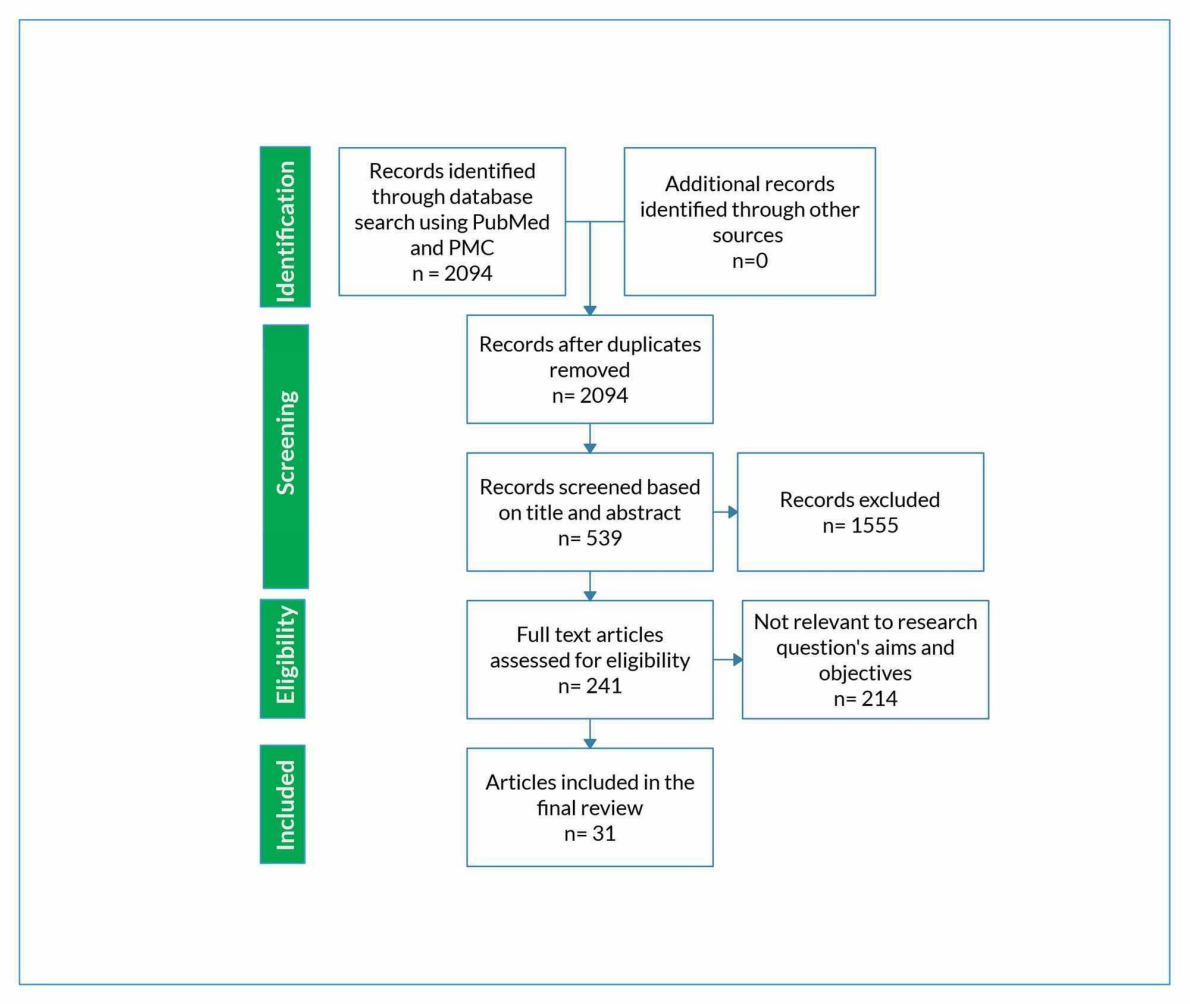
Flowchart depiction ( PRISMA diagram) of the literature review selection procedure PMC, PubMed Central; PRISMA, Preferred Reporting Items for Systematic Reviews and Meta-Analyses

Only those publications that explained the mechanism by which OSA itself poses a risk to cardiovascular health, rather than just the impact of obesity or metabolic syndrome on it, were selected. Figure 1 depicts the Preferred Reporting Items for Systematic Reviews and Meta-Analyses criteria (PRISMA) diagram for the literature review [14].

Discussion

In the analysis of selected data for 21,763 subjects, we found a spectrum of cardiovascular (CV) diseases associated with OSA and the mechanism behind how OSA influences CV events. The detail of these findings is sub-headed as follows to understand them more vividly.

Pathological Changes Seen in OSA

In OSA, the mechanism of forced inspiration against the occluded upper airway produces negative intra-pleural pressure. As the apnea continues, there is a worsening of hypoxia and hypercapnia. Further, hypoxia causes vasoconstriction in the systemic and pulmonary circulation due to sympathetic nervous system activation. A vicious cycle of hypoxia-reoxygenation occurs during the apnea/hypopnea-recovery period that triggers the generation of homocysteine, cysteine, and free radicals, causing oxidative stress [15,16]. Recurrent hypoxia triggers systemic inflammation; there is an alteration in transmural, intrathoracic, and cardiac pressures. The vascular endothelial cell (VEC) injury occurs, which damages the coronary arteries and can lead to coronary artery diseases (CAD) or cardiac ischemia [13,17]. Damage to VECs also occurs due to decreased Nitric Oxide (NO) expression [dysfunction in endothelial NO synthase (eNOS) known as "eNOS uncoupling"] with accelerated superoxide (O_2_−) formation [18].

In a study by Drager et al. [6], we also found that the hypoxia occurring at the cellular level [19] in the adipose tissue leads to lipolysis, macrophage infiltration with chronic inflammation, endoplasmic reticulum strain, and mitochondria damage. Such changes with an additionally observed decrease in adiponectin and an increase in leptin can cause cardio-metabolic dysfunction [20].

OSA is commonly seen in men at a young age with significant changes in their diastolic blood pressure (DBP) and triglyceride (TG) levels. Such changes put men at higher cardiovascular risk than women. Abnormalities in inflammatory cells such as total white blood cells (WBC) or neutrophil counts were typical in age <65 years. However, dysfunction in monocyte or macrophage cells, a precursor for atherosclerotic plaques, were more strongly correlated in women and African Americans with OSA [21]. 

OSA is a leading cause of secondary hypertension. It was observed that OSA patients (asymptomatic mild-to-moderate) who are middle-aged adults have concomitant metabolic syndrome [22], which in turn is believed to be the real cause for high BP. However, OSA independently causes arterial stiffness and non-dipping of blood pressure [23] with alteration of BP circadian rhythm, creating a profound expansion of night-time and morning BP more than the daytime and evening [24]. A clinical trial [10] that had considered possible confounding factors such as BMI, neck circumference, etc. showed a significant increase in DBP, especially the 24-hrs diastolic BP variability (p-value <0.05), as well as an increase in the nocturnal systolic BP (SBP) (p- value<0.05).

A marker for diastolic dysfunction indexed left atrial volume (LAV-i) was significantly altered in OSA patients; these patients were asymptomatic without any symptoms or signs of heart failure (HF). Other changes included the E'/A' ratio (early diastolic mitral flow velocity), E', and A wave (A: atrial contraction) [25]. Patients with nocturnal oxygen saturation level (Spo_2_) <92 independently correlated to E/A ratio, and they had an increased risk of experiencing left ventricular (LV) diastolic dysfunction by almost 2.76-fold. Pressure overload in LV occurs due to the increase in arterial blood pressure, sympathetic activity, and negative intrathoracic pressure. The pulmonary capillary wedge pressure (causing reduced LV compliance) and right ventricular (RV) overdistention (causing LV filling reduction) occurred in the initial stage of OSA [26]. The newly diagnosed patients with severe OSA and an increase in their night-time DBP had a dilated pulmonary artery with main pulmonary artery diameter (MPAd) of 25mm or higher [27].

OSA increases both the systolic and diastolic BP [28], and there is a frequent occurrence of nocturnal ventricular ectopy, especially in subjects with respiratory event index (REI) >15/h presenting with stable acute heart failure. Arterial hypertension causes loss of cerebral vascular autoregulation [29]. With recurrent hypoxia seen in OSA, there is a higher risk of the silent cerebral infarct, commonly in the lacunar regions [12]. Young men with high BMI, approximately cover 14% of the study subject [30], who presented with acute type A aortic dissection, had underlying sleep apnea and hypertension.

One clinical trial [31] showed an increase in cortisol levels by 15-20% in OSA. Increased cortisol level with chronic activation of the hypothalamic-pituitary-adrenal (HPA) axis causes traditional cardiac risks such as hypertension, diabetes, and the central deposition of fat [32]. The study also showed changes in sleep stages: an increase in Stage 1 and a decrease in Stage 2 of sleep. Women in the study scored more on the Beck Depression Inventory-II (BDI-II), though clinical depression or its diagnosis could not be concluded.

Cardiovascular Biomarkers in OSA

Repeated hypoxia observed in OSA causes the release of various proteins that leads to VEC injury, a precursor for cardiovascular diseases. These proteins can serve as biomarkers for diagnosing the severity of cardiovascular diseases, even in asymptomatic OSA patients. Detail of such proteins is given in Table 2.

**Table 2:** The proteins involved in the pathogenesis of cardiovascular diseases seen in OSA OSA, Obstructive Sleep Apnea; SBP, Systolic Blood Pressure; DBP, Diastolic Blood Pressure; AHI, Apnea-Hypopnea Index; LOS, Lowest Oxygen Saturation; VEC, Vascular Endothelial Cell; ODI, Oxygen Desaturation Index; AT-1, Angiotensin-1; AT-2, Angiotensin-2

Reference	Study Design		Year of publication	Sample size (n)	Finding	Comments
Kun Li et al. [33]		Clinical trial	2019	157	Protein YKL-40	Protein YKL-40 was found to be higher in OSA subjects, especially with hypertension. The expression of this protein was significantly associated with SBP, DBP, AHI, and LOS. This protein is involved in inflammation, migration of cells, and tissue remodeling. These causes VEC injury and promote atherosclerosis.
Shuhui Wang et al. [2]		Observational	2018	35	Matrix Metalloproteinase-9 (MMP-9)	Hypoxia in OSA leads to the release of MMP-9 protein, which leads to VEC injury via the hypoxia-MMP-9-β_2_AR (beta2-adrenergic receptor) signaling axis.
Xiuping Yang et al. [34]		Observational	2018	60	Protein miRNA dysregulation	Dysregulated miRNA proteins were seen in OSA patients, possibly targeting genes involved in the metabolism and regulation of endothelial cells.
Macy M S Lui et al. [35]		Observational	2018	98	High-sensitivity troponin l (hsTnI) and C-Reactive Protein (CRP)	Newly diagnosed asymptomatic patients with OSA had increased levels of hsTnI and CRP, depending on their AHI and ODI that represented stable or subclinical cardiac injury and the role of inflammation in VEC injury, respectively.
Rami N Khayat et al. [18]			2018	21	AT-1 and AT-2 expressions	Upregulation of AT-1 and AT-2 is observed in VEC injury. OSA subjects with no-to-minimum cardiovascular risk were found to have increased expression of AT-1 and AT-2. Such changes could be the major contributing factor for cardiovascular disease.
Vahid Mohsenin et al. [36]		Observational	2011	22	Soluble fms-like tyrosine kinase-1 (sFlt-1) and soluble endoglin (sEng)	sFlt-1 and sEng are antiangiogenic proteins that cause endothelial dysfunction. It was increased in severe OSA in response to hypoxic stress, especially in patients with hypertension.

Effect of OSA Treatment on Cardiovascular Diseases

A single-blinded clinical trial [37] for hypertensive patients with severe OSA (AHI >30) received a titrated dose of antihypertensive medications to which CPAP therapy was added after three weeks. Subjects received crossed-over treatment with CPAP: effective (4-15 mm H_2_0) and placebo (4 mm H_2_O) CPAP. It was seen that patients on effective CPAP had decreased carotid-femoral pulse wave velocity (cfPWV) of 0.7 ± 0.6 m/sec (with a p-value of = 0.03), achieving their target cfPWV by a near increase of 1.5 times more. The above effect was comparable to the results seen in patients on lipid-lowering agents [38]. A decrease in cfPWV of 1.6 m/sec could add 15 years of functional rejuvenation to the vessels.

In the clinical trial by Walia et al. [39] factors like BMI, smoking, and diabetes mellitus that directly affect BP control were adjusted. These subjects had uncontrolled BP despite taking an effective anti-hypertensive drug regime. The study showed OSA through pharmacokinetics, and chrono-therapeutic influence, activates a more drug-resistant hypertensive pathway. They concluded that severe untreated OSA could lead to resistant hypertension (nearly by four-fold more odds) by additional interference with antihypertensive medications' effectiveness. Lack of medication adherence is commonly seen in symptomatic OSA patients. Fatigue, sleepiness, or impaired concentration can hamper a person's memory and decision-making skills. Hence, OSA's treatment offers cardio-protective benefits as well as improvement in medication adherence with better control of BP [40].

In one study [31], patients receiving randomized CPAP therapy had improved 24-hour cortisol level leading to the reversal of hypercortisolemia (no such effect was observed on the circadian cortisol level).

Miscellaneous Conditions Associated with OSA

The presence of OSA and chronic kidney disease (CKD) can mutually enhance the magnitude of each other's problems [41], especially in the setting of end-stage renal disease (ESRD) [42]. They may synergistically act to modify the prognosis or precipitate numerous cardiovascular disorders. To know the clinical significance of such effects requires further research in the future. Table 3 shows other common conditions seen in OSA patients.

**Table 3:** Common conditions associated with OSA and its medical significances OSA, Obstructive Sleep Apnea; CKD, Chronic Kidney Diseases; HR, Hazard Ratio; CI, Confidence Interval

Reference	Study Design	Year of publication	Sample size (n)	Finding
Ronaldo D Piovezan et al. [43]	Observational	2017	657	Vitamin D deficiency was seen in 59.5 % of subjects with OSA (moderate OSA with p-value <0.01 and severe OSA with p-value =0.03) and short sleep duration (<6hours, with p-value =0.01). An Independent link was established between these two conditions that were more commonly seen in African Americans ethnicity, female, obese, smokers having a sedentary lifestyle, hypertension, and diabetes.
Yu-Sheng Lin et al. [44]	Observational	2017	6866	OSA patients were at increased risk for developing CKD, median period of 3.2 years; HR was 1.37 (95 % CI, 1.05-1.77; p-value = 0.019). CKD occurred approximately 2.5 months earlier than in the patients without OSA. The correlation was observed after adjusting for hypertension and diabetes. The relation was most robust among women.
Josef Yayan et al. [41]	Observational	2017	382	CKD develops more often in patients with OSA than in non-OSA. Almost 70% of OSA subjects developed CKD, whereas only 36% of non-OSA subjects developed CKD.

Strengths and limitations

This literature review has mostly included observational studies and clinical trials. No meta-analysis was included. The majority of the selected studies have explained the independent effect of OSA on the cardiovascular system. However, a minority of them have also explained the co-existing effects of metabolic syndrome and obesity on OSA and CV health. Other drawbacks of this study are using a limited electronic database (PubMed and PMC) and excluding non-English literature. For this article, some of the points like definition have been quoted from other references in the original article, which dates more than 10 years back.

## Conclusions

In this review, we found distinct, independent effects of OSA on the cardiovascular system. The recurrent intermittent hypoxia occurring at the cellular level in OSA provokes neuro-humoral and biochemical changes. This includes sympathetic nervous system activation, systemic inflammation with oxidative stress, changes in lipid profile, VEC injury with dysfunction, and activation of the renin-angiotensin-system (RAS) and hypothalamic-pituitary-axis (HPA). These changes occur very early in an OSA patient, independent of the AHI events and OSA severity. Thus, OSA is linked with various cardiovascular changes/diseases as follows: an increase in arterial BP (DBP>SBP; resistant hypertension was prevalent), atherosclerotic changes in the coronary and cerebral vasculature (CAD, ischemic stroke), left ventricular diastolic dysfunction (heart failure), alteration in the main pulmonary artery caliber, and the right ventricular system dysfunction. Successful reversal of such neuro-humoral and biochemical expressions is observed with CPAP treatment. OSA can be easily detected with polysomnography/sleep studies, which may not be cost-effective and convenient to perform in all patients. However, a high level of clinical suspicion should be present to detect OSA, especially in the setting where the cardiovascular disease is uncontrolled while on its usual treatment. OSA screening can be done simply by asking for symptoms such as snoring, choking, fatigue, or excessive daytime sleepiness. Annual polysomnography can be advised for screening OSA in suspected patients, as it may be better to over-diagnose than to underdiagnose an ominous disorder. Future research activities are required to determine when polysomnography as a screening tool for OSA can effectively improve cardiovascular outcomes.
